# ﻿Three new species of the genus *Metapocyrtus* Heller 1912, subgenus *Orthocyrtus* Heller 1912 (Coleoptera, Curculionidae, Entiminae, Pachyrhynchini), from Mindanao Island, Philippines

**DOI:** 10.3897/zookeys.1088.79021

**Published:** 2022-03-09

**Authors:** Analyn Anzano Cabras, Milton Norman Medina, Genelyn Madjos, Maurizio Bollino

**Affiliations:** 1 Coleoptera Research Center, University of Mindanao, Davao City, Philippines University of Mindanao Davao City Philippines; 2 Department of Biological Sciences, College of Science and Mathematics, Western Mindanao State University, Zamboanga City, Philippines Western Mindanao State University Zamboanga City Philippines; 3 Museo di Storia naturale del Salento, 73021 Calimera, Lecce, Italy Museo di Storia Naturale del Salento Lecce Italy

**Keywords:** Archipelago, beetles, endemic, novel species, taxonomy, weevils

## Abstract

Three new species of genus Metapocyrtus Heller, 1912, subgenus Orthocyrtus Heller, 1912 (Coleoptera, Curculionidae, Entiminae, Pachyrhynchini) from Mindanao Island, Philippines are described: M. (O.) regalis**sp. nov.**, M. (O.) tboli**sp. nov.**, and M. (O.) reagani**sp. nov.** Photographs of their habitus and male genitalia are presented.

## ﻿Introduction

The genus *Metapocyrtus* Heller, 1912 is a hyperdiverse member of the tribe Pachyrhynchini exclusively distributed in the Philippine islands. It is set apart from other members of the tribe by these general diagnostic characters: “rostrum apically not swollen, basally with a more or less strongly pronounced transverse groove, eyes moderately convex, not bulging, scape of antenna reaching at least to or beyond hind margin of eye” (Schultze 1925: 135). Currently, it includes more than 230 described species and seven subgenera, but with many taxa remaining still undescribed (Yap and Gapud 2008; Yoshitake 2017; Bollino et al. 2020; Cabras and Medina 2021). One of the subgenera of *Metapocyrtus* is *Orthocyrtus* Heller, 1912, whose members are conspicuous for their large size. At present there are 39 *Orthocyrtus* species described, which are distributed all over the Philippines (Cabras et al. 2021a, 2021b).

In Mindanao, five new species of *Orthocyrtus* have recently been described—M. (O.) mansaka Cabras, Bollino & Medina, 2018, M. (O.) ginalopezae Cabras & Medina, 2019, M. (O.) davaoensis Cabras, Medina & Bollino, 2021, M. (O.) hirakui Cabras, Medina & Bollino, 2021, and M. (O.) barsevskisi Cabras, Villanueva & Medina, 2021 (Cabras et al. 2018, 2021a, 2021b; Cabras and Medina 2019)—of which three are known from Davao region, one from Bukidnon, and another one from Surigao. The discovery of new species from Mindanao comes as no surprise as it is one of the remaining frontiers of biodiversity in the Philippines and coleopterological work on this island has been limited. As a result of our sampling, and thanks to several donations from friends and colleagues, we have had the opportunity to identify some specimens as belonging to species new to science; of these, three are described and illustrated in this article.

## ﻿Materials and methods

The specimens deposited in the University of Mindanao Coleoptera Research Center were collected by sheet beating and hand picking and killed in vials with ethyl acetate. Morphological characters were observed under Luxeo 4D and Nikon SMZ745T stereomicroscopes. The illustrations, as well as the treatment of the genitalia, followed that of Yoshitake (2011). Due to the little or almost no use of the chitinous structures of the female genitalia in identifying and characterizing the species of Pachyrhynchini (Bollino et al. 2020), these anatomical parts are no longer illustrated. Images of the habitus and genitalia were taken using a Nikon D5300 digital camera with a Sigma 18–250 macro lens. All images were stacked and processed using a licensed version of Helicon Focus 6.7.0 and Photoshop CS6 Portable software. Label data are indicated verbatim. Abbreviations and symbols mentioned in this paper are abbreviated as follows:

/ different lines;

 // different labels;

**ā** arithmetic mean;

**LB** length of the body in dorsal view, from the apical margin of the pronotum to the apices of the elytral;

**LE** length of the elytra in dorsal view, from the level of the basal margins to the apices of the elytral;

**LP** length of the pronotum, from the base to apex along the midline;

**LR** length of the rostrum

**WE** maximum width across the elytra;

**WP** maximum width across the pronotum;

**WR** maximum width across the rostrum.

All measurements are in millimeters.

Comparative materials and specimens used in the study are deposited in the following institutional collections:

**MBLI** private collection of Maurizio Bollino, Lecce, Italy;

**SMTD** Senckenberg Natural History Collections, Dresden, Germany;

**PNM**Philippine National Museum of Natural History, Manila, Philippines;

**UMCRC** University of Mindanao Coleoptera Research Center, Davao City Philippines.

## ﻿Taxonomy

### ﻿ Metapocyrtus (Orthocyrtus) regalis

Taxon classificationAnimaliaColeopteraCurculionidae

Cabras, Medina & Bollino sp. nov.

4216AE18-A671-5D32-A520-C548A74645E1

http://zoobank.org/01D13D5B-F320-416B-BDE3-0AAE170B61E9

Figs 1–4

#### Holotype

**Figures 1–4.**
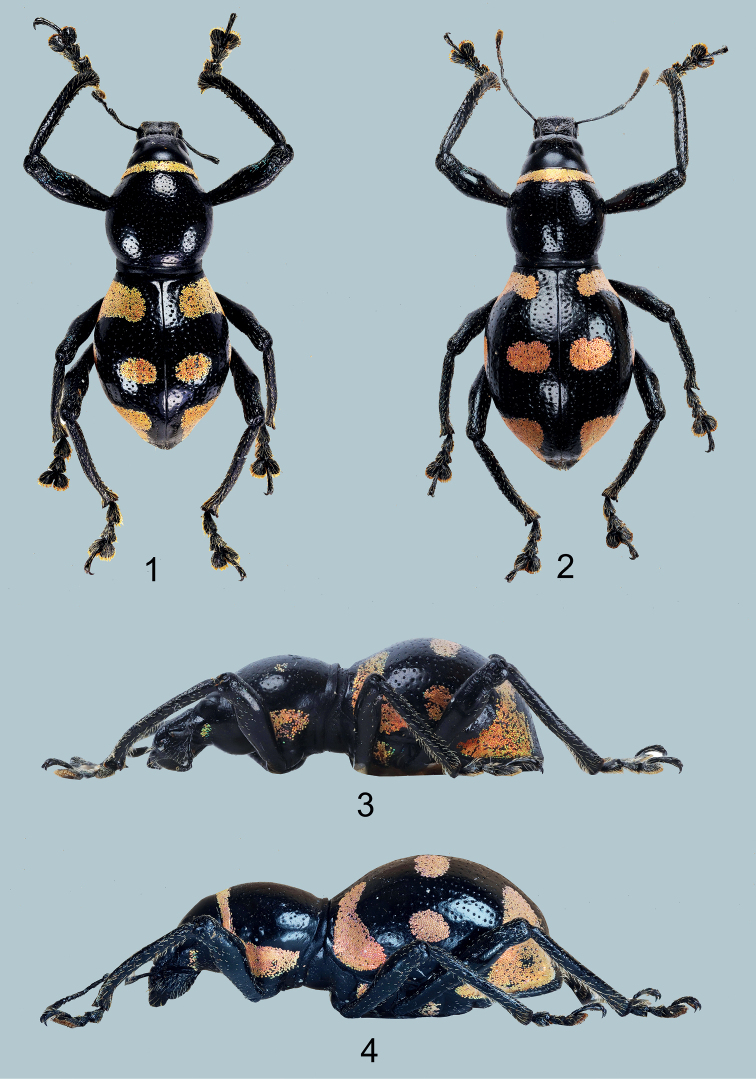
Metapocyrtus (Orthocyrtus) regalis sp. nov. **1** male holotype, dorsal view **2** female, dorsal view **3** ditto, male, lateral view **4** ditto, female, lateral view.

(Figs 1, 3), male: Philippines – Mindanao / Gutalac / Zamboanga del Norte / Nov. 2021/ coll. Local collector (typed on white card) // Holotype male / Metapocyrtus (Orthocyrtus) regalis / Cabras, Medina & Bollino, 2021 (typed on red card). Presently in UMCRC, will be deposited in PNM.

#### Paratypes

(21 ♂♂, 18 ♀♀): 3 ♂♂, 3 ♀♀: same data of the holotype, all in UMCRC; 1 ♂: Philippines – Mindanao / Gutalac / (Zamboanga del Norte) / IX–XI.2016 / coll. Bollino; 2 ♂♂, 5 ♀♀: Philippines – Mindanao / Gutalac / (Zamboanga del Norte) / VII.2018 / coll. Bollino;1 ♂: Philippines – Mindanao / near Siocon / (Zamboanga del Norte) / VII–IX.2019 / coll. Bollino; 13 ♂♂, 9 ♀♀: Philippines – Mindanao Island / Sitio Quary, Barangay Donia Cecilia / Sirawai (Siocon, Zamboanga del Norte) / ~ 7°34'N, 122°10'E - m 100 – IX.2018 / Lgt. local people - coll. Bollino, all in MBLI; 1 ♂, 1 ♀: Philippines – Mindanao Island / Sitio Quary, Barangay Donia Cecilia / Sirawai (Siocon, Zamboanga del Norte) / ~ 7°34'N, 122°10'E - m 100 – IX.2018 / Lgt. local people - coll. Bollino, will be deposited in SMTD.

#### Diagnosis.

Metapocyrtus (Orthocyrtus) regalis sp. nov. is different from all other species of *Orthocyrtus* by the following features: robust, moderately stout, strongly convex body and yellowish-orange elytral markings consisting of a thin band from stria II to lateral margin, widened laterally; two median subcircular patches from stria I–IV and from stria VI–IX, and a subtriangular patch on apical third from stria II to lateral margin.

#### Description.

**Male.** Dimensions (in mm): LB 11.8–12.5 (holotype 11.8, ā: 12.03), LR 2.2–2.1 mm (2.2, ā: 2.26), WR 1.9–2.0 (1.9 mm, ā: 1.96), LP 4.5–5.0 (4.5, ā: 4.67), WP: 4.5–5.0 (4.5, ā: 4.67), LE 7.0–7.8 (7.5, ā: 7.43), WE 5.5–6.0 (6.0 mm, ā: 5.83). *N* = 4.

Integument black. Body surface, rostrum, head, and underside lustrous.

***Body*** subglabrous.

***Head*** subglabrous; dorsal surface smooth but lateroventral surface with sparse minute and light-colored hairs; forehead between eyes slightly bulging with faint longitudinal groove almost reaching the vertex. Eyes medium-sized and feebly convex; lateral sides below the eyes with sparse, light-yellow, round to elliptical scales.

***Rostrum*** rugose on basal half and finely punctured on apical half, longer than wide (LR/WR: 1.16), bearing minute, light-colored, adpressed hairs on dorsum and long, whitish hairs on lateral surface; transverse basal groove distinct; longitudinal groove along midline distinct with a shallow, pit-like depression; dorsum weakly convex dorsally; lateral sides with weakly widened apicad. Antennal scape slightly shorter than funicle, moderately covered with fine, light-colored hairs. Funicular segments I and II almost of the same length, twice as long as wide; segments III–VII nearly as long as wide; club subellipsoidal, nearly three times longer than wide.

***Prothorax*** subglobular, as long as wide (LP/WP: 1.0), moderately punctate with minute hairs, widest at middle, sides evenly arcuate, strongly convex, and with the following scaly markings of yellowish-orange, round scales with a pink to purple shimmer: a) thin band at the anterior margin and b) broad lateroventral stripe before the coxa confluent with the anterior band.

***Elytra*** subovate (LE/WE:1.25), wider and nearly twice as long as prothorax (WE/WP: 1.33, LE/LP: 1.67); body surface black, subglabrous, setiferous punctate, moderately stout; dorsum strongly and uniformly convex in profile with a gradual apical declivity; apex with sparse, light-colored, fine hairs. Each elytron with the following scaly markings of yellowish-orange, round scales with a pink to purple shimmer: a) thin band from stria II to lateral margin, slightly constricted on stria V then widened laterally, b) two subcircular median patch from stria I–IV, and from stria VI–IX, and d) subtriangular patch on apical third from stria II to lateral margin.

***Legs*** with strongly clavate femora. Femora sparsely covered with minute, light-colored hairs and moderately covered with minute, blue-green, elliptical scales towards apical quarter. Tibiae covered with subadpressed, light-colored hairs and long, light-colored bristles along inner margin; tibiae weakly serrate along inner margin. Fore tibiae bear a mucro at apex. Tarsomeres covered with pubescence. Coxae barely pubescent with pale, bluish to yellowish, round scales on distal end. Mesoventrite covered with light-colored, adpressed hairs. Metaventrite with light-colored, adpressed hairs and yellowish, round scales at distal ends. Ventrite I weakly depressed on disc, with light-colored, adpressed hairs and yellowish to bluish, round scales towards distal ends. Ventrites II–V sparsely covered with adpressed hairs, especially towards margin. Ventrite V flattened, apical half finely densely punctured, interspersed sparsely with light-colored hairs.

***Male genitalia*** as shown in Figs 13–15.

**Female.** Dimensions (in mm): LB 12.0–14.8 (ā: 13.4), LR 2.0–2.5 (ā: 2.25), WR 1.7–1.9 (ā: 1.8). LP 3.7–4.0 (ā: 3.85). WP 4.0–4.5 (ā: 4.25), LE 9.0–9.1 (ā: 9.05). WE 6.0–7.0 (ā: 6.5). *N* = 3.

Habitus as shown in Figs 2, 4.

Females differ from males in the following: a) pronotum (LP/WP 0.88-0.93), slightly shorter than in male; b) pronotum less arcuate than male, and c) elytra imperfectly subovate (LE/WE 1.27–1.3), longer and wider (WE/WP 1.64–1.67, LE/LP 2.97–3.0) than in male; d) ventrite I flattened, less hairy and not depressed on disc. Otherwise female similar to the male.

#### Etymology.

The specific epithet comes from the Latin adjective *regalis* (royal, regal), which refers to the regal appearance of this species deriving from its bright yellowish-orange coloration.

#### Distribution.

Metapocyrtus (Orthocyrtus) regalis sp. nov. is known from Zamboanga del Norte (Fig. 22).

### ﻿ Metapocyrtus (Orthocyrtus) tboli

Taxon classificationAnimaliaColeopteraCurculionidae

Cabras, Medina & Bollino sp. nov.

0D00E141-66BE-518C-B39A-11F3FB53B656

http://zoobank.org/30B4B166-D34E-4227-9ED4-D74027233BBC

Figs 5–8

#### Holotype

**Figures 5–8.**
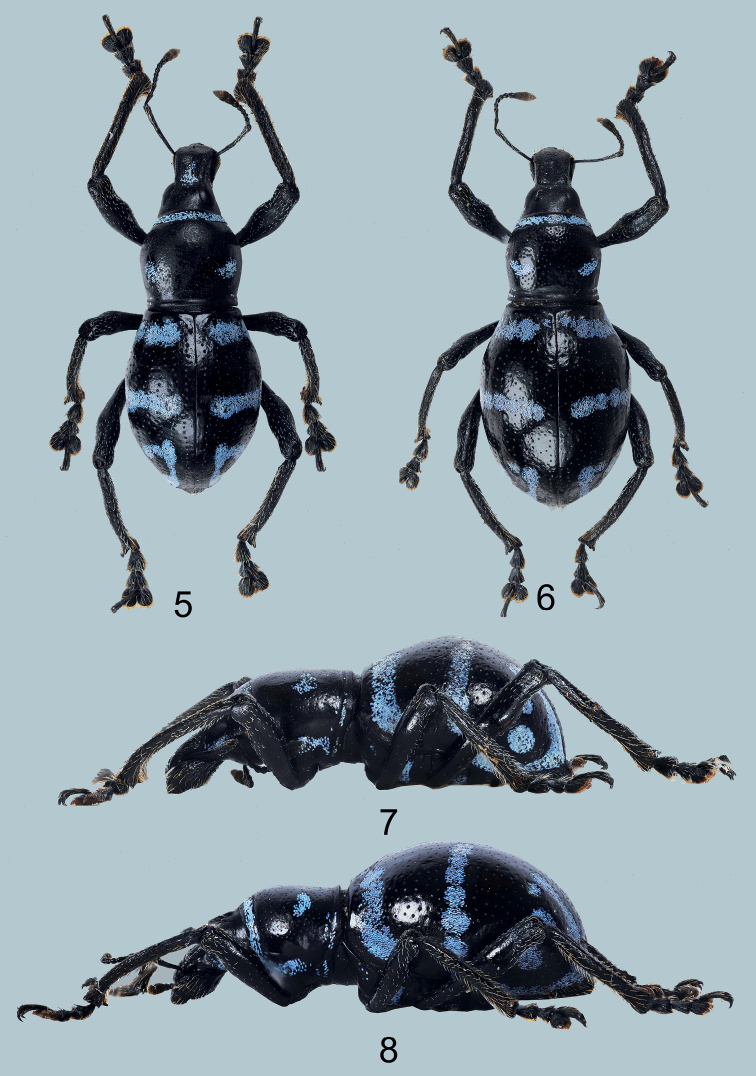
Metapocyrtus (Orthocyrtus) tboli sp. nov. **5** male holotype, dorsal view **6** female, dorsal view **7** ditto, male, lateral view **8** ditto, female, lateral view.

(Figs 5, 7), male: Philippines – Mindanao / Kapatagan / Davao del Sur / Nov. 2020 / coll. Local collector (typed on white card) // HOLOTYPE male / Metapocyrtus (Orthocyrtus) tboli/ CABRAS, MEDINA & BOLLINO, 2021 (typed on red card). Presently in UMCRC, will be deposited in PNM.

#### Paratypes

(55♂, 32♀♀): 1♂, 2♀♀: same data of the Holotype, all in UMCRC; 48 ♂♂, 29 ♀♀: Philippines – Mindanao / Davao – Mt. Apo / VIII.2009 – m 1000–1200 / lg. local people – coll. M. Bollino; 1 ♂: Philippines – Mindanao / Sarangani / Kiamba / IX.2015 / coll. Bollino; 2 ♂♂: Philippines – Mindanao / Kiamba / (Sarangani) / January–March 2071 / coll. Bollino; 1 ♂: Philippines – Mindanao / Mt. Kapatagan / Davao del Sur Prov. / IX–X.2012 / coll. Bollino; 1 ♂: Philippines – Mindanao / Mt. Apo – Calatagan / (Davao del Sur-Brgy. Digos) / VI–VII.2017 – m 1400–1500 / coll. Bollino, all in MBLI; 1 ♂, 1 ♀: Philippines – Mindanao / Davao – Mt. Apo / VIII.2009 – m 1000-1200 / lg. local people – coll. M. Bollino, will be deposited in SMTD.

#### Diagnosis.

Metapocyrtus (Orthocyrtus) tboli sp. nov. differs from all other species of *Orthocyrtus* by having its elytral scaly marking consisting of a thin, subbasal, median transverse stripe from stria I towards the lateral margin, a longitudinal stripe on apical third along stria II, a long postmedian stripe along the lateral margin towards apex, which is confluent with the stripe along stria II, and two small dots on intervals V and VI on the apical quarter, at times confluent with the distorted longitudinal stripe on stria II and forming a subtriangular shape.

#### Description.

**Male.** Dimensions (in mm): LB 11.0–11.1 (holotype 11.0, ā: 11.05), LR 2.2–2.5 m (2.2, ā: 2.35), WR 1.8–2.0 (1.8 mm, ā: 1.9), LP 3.7–3.9 (3.7, ā: 3.8), WP: 4.0–4.1 (4.0, ā: 4.05), LE 7.0–7.1 (7.0, ā: 7.05), WE 5.5–5.8 (5.5 mm, ā: 5.65). *N* = 2.

Integument black. Body surface, rostrum, head, and underside with weak luster.

***Body*** subglabrous.

***Head*** subglabrous with sparse and minute pubescence on lateroventral side; underside of eyes with elongated stripe of metallic, light blue, elliptical scales interspersed with subadpressed setae; frons in between eyes nearly flattish with sparse, light-blue, round scales. Eyes medium-sized and moderately convex.

***Rostrum*** finely punctured, longer than wide (LR/WR: 1.22), bearing sparse, minute, light-colored, adpressed hairs on dorsum; lateral surface covered with long, whitish hairs; anterolateral sides with long, yellowish hairs; transverse basal groove distinct; longitudinal groove along midline visible with shallow depression beset with light-blue, minute, round scales; dorsum weakly convex dorsally; lateral sides with moderately widened apicad. Antennal scape as long as the funicle, moderately covered with fine, light-colored hairs, especially toward the distal ends. Funicular segments I and II almost of the same length, twice as long as wide; segments III–VII nearly as long as wide; club subellipsoidal, nearly three times longer than wide.

***Prothorax*** subglobular, slightly wider than long (LP/WP: 0.925), with fine, setiferous punctures, widest before middle with highest point just after apical margin, weakly convex, and with the following scaly markings of minute, light-blue, round scales: a) fine transverse stripe along the anterior margin, b) thin lateroventral band before the coxa confluent with the anterior band, c) two small subcircular spots on middle of discs, and d) thin stripe along the lateral side of posterior margin.

***Elytra*** subovate (LE/WE:1.27), significantly longer and moderately wider than prothorax (WE/WP: 1.38, LE/LP: 1.89); body surface black, subglabrous, setiferous punctate, strongly convex dorsally; apex with sparse, light-colored, fine hairs. Each elytron with the following scaly markings of minute, light-blue, round scales: a) a thin, subbasal, transverse stripe from stria I towards lateral margin, b) a thin, median, transverse stripe starting from interval I towards but not reaching lateral margin, c) a longitudinal stripe on apical third along stria II, d) a long postmedian stripe along lateral margin extending towards apex, confluent with the longitudinal stripe on stria II, and d) two small dots on interval V and VI on apical quarter, at times confluent with the distorted longitudinal stripe on stria II, forming a subtriangular shape.

***Legs*** with strongly clavate femora. Femora sparsely covered with minute, light-colored setae, denser towards the apex. Tibiae covered with subadpressed, brownish hairs and long, light-colored bristles along inner margins; tibiae weakly serrate along inner margin. Fore tibiae bear a mucro at apex. Tarsomeres covered with pubescence. Coxae barely pubescent with light-coloured, elliptical scales and light-colored hairs on distal end. Mesoventrite covered with light-colored, adpressed hairs. Metaventrite depressed with light-colored, adpressed hairs and light-blue, round scales at sides. Ventrite I moderately depressed on disc, with light-colored, adpressed hairs and light-blue, round scales towards distal ends. Ventrites II–V sparsely covered with adpressed hairs, especially towards margin. Ventrite V flattened, apical half finely densely punctured, interspersed sparsely with light-coloured hairs.

***Male genitalia*** as shown in Figs 16–18.

**Female.** Dimensions (in mm): LB 10.5–11.5 (ā: 11), LR 2.0–2.1 (ā: 2.05), WR 1.6–1.8 (ā: 1.7). LP 3.0–3.5 (ā: 3.25). WP 3.8–4.0 (ā: 3.9), LE 7.5–8.1 (ā: 7.8). WE 5.8–6.5 (ā: 6.15). *N* = 2.

Habitus as shown in Figs 6, 8.

Females differ from males in the following: a) pronotum (LP/WP 0.79-0.88), slightly shorter than in male; b) pronotum less arcuate than male, and c) elytra imperfectly subovate (LE/WE 1.25–1.29), longer and wider (WE/WP 1.52–1.63, LE/LP 2.31–2.5) than in male, widest before middle; d) ventrite 1 flattened, less hairy, and not depressed on disc. Otherwise female similar to the male.

#### Etymology.

The new species is named after the indigenous community inhabiting Kiamba Saranggani, the Tboli people. It is a noun in apposition.

#### Distribution.

Metapocyrtus (Orthocyrtus) tboli sp. nov. is known from Saranggani and Davao provinces (Fig. 22).

### ﻿ Metapocyrtus (Orthocyrtus) reagani

Taxon classificationAnimaliaColeopteraCurculionidae

Cabras, Medina & Bollino sp. nov.

7969BE5E-764C-531C-A8CB-BF55C530EEA9

http://zoobank.org/4E203273-7F1C-461E-B304-8C98E24594EA

Figs 9–12

#### Holotype

**Figures 9–12.**
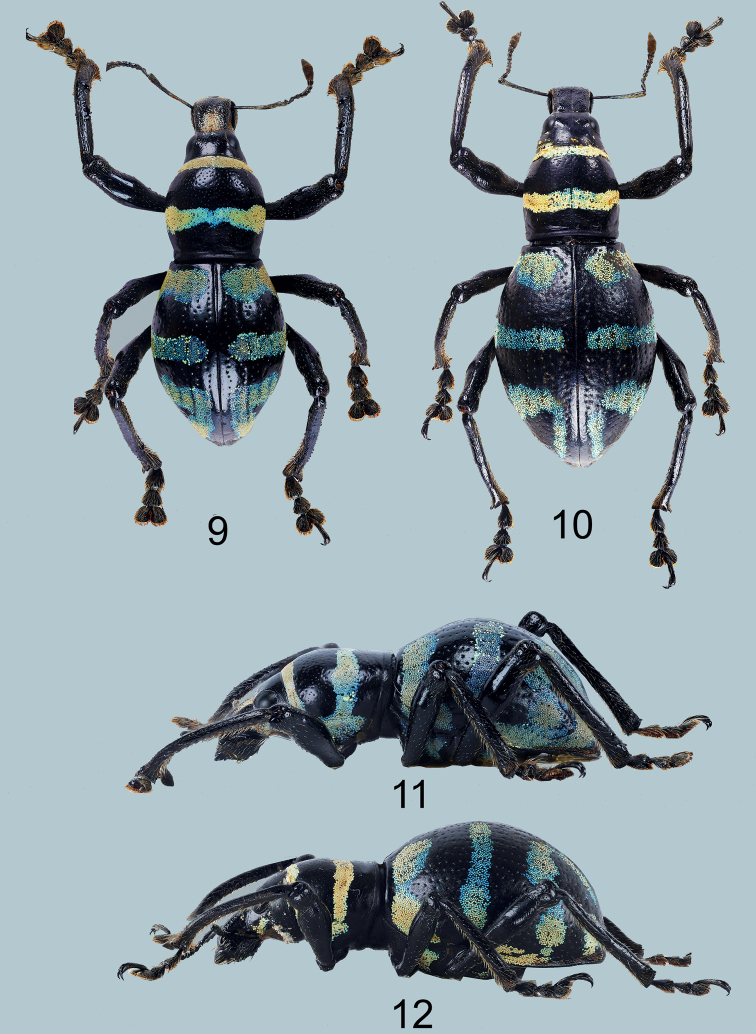
Metapocyrtus (Orthocyrtus) reagani sp. nov. **9** male holotype, dorsal view **10** female, dorsal view **11** ditto, male, lateral view **12** ditto, female, lateral view.

(Figs 9, 11), male: Philippines – Mindanao / Talakag / Bukidnon / July 2018 / coll. Reagan Joseph Villanueva (typed on white card) // HOLOTYPE male / Metapocyrtus (Orthocyrtus) reagani / CABRAS, MEDINA & BOLLINO, 2021 (typed on red card). Presently in UMCRC, will be deposited in PNM.

#### Paratypes

(8♂♂, 8♀♀): 6♂♂, 5♀♀: same data of the Holotype, all in UMCRC; 2♂♂, 2 ♀♀: Philippinen: / N-Mindanao Insel / Mt. Kitanglad / IV.1987; 1 ♀: Philippines – Mindanao / Intavas / (Bukidnon) / January–March 2016 / coll. Bollino, all in MBLI.

#### Diagnosis.

Metapocyrtus (Orthocyrtus) reagani sp. nov. differs from all other species of the subgenus by its unique elytral marking consisting of thick basal bands, a moderately thin median transverse band, and a distorted subtriangular stripe on apical third, and by its stout aedeagus. Contrary to M. (O.) tboli, which has two small spots on each side of the disc, M. (O.) reagani has a transverse median stripe on the pronotum.

#### Description.

**Male.** Dimensions (in mm): LB 12.5–13.0 (holotype 12.5, ā: 12.71), LR 2.0–2.2 m (2.0, ā: 2.1), WR 1.6–1.9 (1.6 mm, ā: 1.78), LP 4.0–4.1 (4.0, ā: 4.03), WP: 4.7–5.0 (4.7, ā: 4.86), LE 8.0–8.5 (8.0, ā: 8.3), WE 6.0–6.5 (6.0 mm, ā: 6.24). *N* = 7.

Integument black. Body surface, rostrum, head, and underside with a weak luster.

***Body*** subglabrous.

***Head*** subglabrous, sparsely minutely pubescent, with elongated stripe of metallic, light-yellow and pale-blue, elliptical to round scales on each side below the eye, and light-colored hairs on lateroventral parts; forehead between eyes with sparse metallic, light-yellow and pale-blue, round scales. Eyes medium-sized and moderately convex.

***Rostrum*** weakly rugose and coarsely punctured, longer than wide (LR/WR: 1.25), bearing minute, light-colored, adpressed hairs on dorsum; lateral surface below antennal scrobes moderately covered with short, brownish hairs, which become longer towards apex; transverse basal groove distinct; longitudinal groove along midline faint, slightly depressed on middle and beset with metallic, light-yellow and pale-blue, round scales; dorsum finely punctured; dorsal surface moderately convex; lateral sides with weakly widened apicad. Antennal scape slightly shorter than funicle, moderately covered with fine, light-colored hairs. Funicular segments I and II almost of the same length, twice as long as wide; segments III–VII nearly as long as wide; club subellipsoidal, nearly three times longer than wide.

***Prothorax*** subglobular, slightly wider than long (LP/WP: 0.85), finely punctate, widest at middle, weakly convex, and with the following scaly markings of metallic, light-yellow and pale-blue, round scales: a) fine transverse band at the anterior margin, b) transverse band in the entire width in the middle, and c) broad lateroventral stripe before the coxa confluent with the anterior and medial bands.

***Elytra*** subovate (LE/WE:1.23), slightly wider and twice longer than prothorax (WE/WP: 1.28, LE/LP: 2.0), body surface black, sub-glabrous, finely setiferous punctate, moderately convex; apex with sparse, light-colored, fine hairs. Each elytron with the following scaly markings of metallic, light-yellow to pale-blue, round scales: a) a thick transverse band from stria I to lateral margin slightly constricted on stria V; b) a thin, median, transverse band starting from stria I to stria VIII; c) a distorted subtriangular stripe on apical third; d) a long stripe along lateral margin from behind the middle to apex, confluent with apical stripes.

***Legs*** with strongly clavate femora. Femora sparsely covered with minute, light-colored hairs, with apex sparsely covered with pale-blue, hair-like scales. Tibiae covered with subadpressed, light-colored hairs and long, light-colored bristles along inner margin; tibiae weakly serrate along inner margin. Fore tibiae bear a mucro at apex. Tarsomeres covered with pubescence. Coxae barely pubescent with yellow ochre, round scales on distal end. Mesoventrite covered with light-colored, adpressed hairs. Metaventrite with light-colored, adpressed hairs and light-yellow ochre, round scales at lateral sides. Ventrite I weakly depressed on disc, with light-colored, adpressed hairs and light-yellow ochre, round scales towards lateral margin. Ventrites II–V sparsely covered with adpressed hairs, especially towards margin. Ventrite V flattened, apical half finely densely punctured, interspersed sparsely with pale-blue, hair-like scales. Ventrites I–V with dense and long, light-brown hairs; laterally with sparse, light-colored hairs.

***Male genitalia*** as shown in Figs 19–21.

**Female.** Dimensions (in mm): LB 14.5–14.8 (ā: 14.62), LR 2.5–2.6 (ā: 2.54), WR 2.0–2.2 (ā: 2.08). LP 4.0–4.2 (ā: 4.08). WP 5.0–5.2 (ā: 5.08), LE 10.0–10.3 (ā: 10.12). WE 8.0–8.3 (ā: 8.12). *N* = 5.

**Figures 13–21.**
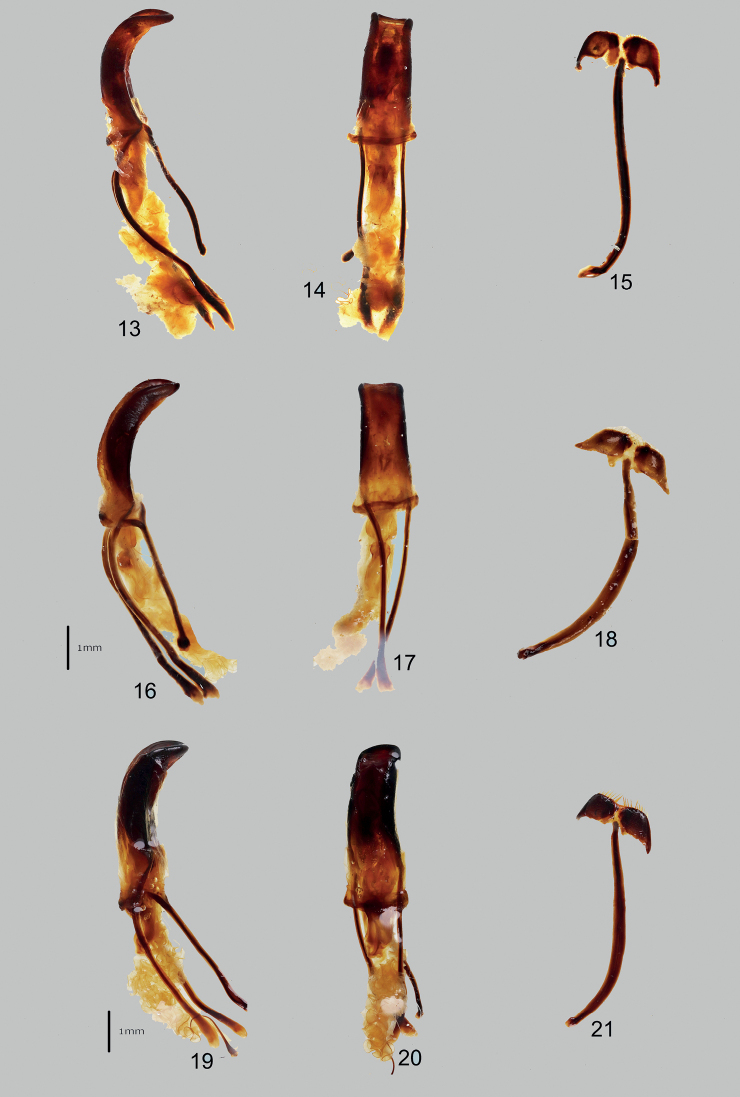
Male genitalia of *Orthocyrtus* species **13–21**: **13–15**Metapocyrtus (Orthocyrtus) regalis sp. nov. **16–18**Metapocyrtus (Orthocyrtus) tboli sp. nov. **19–21**Metapocyrtus (Orthocyrtus) reagani sp. nov.; **13, 16, 19** aedeagus in lateral view **14, 17, 20** idem. in dorsal view **15, 18, 21** sternite IX in dorsal view.

Habitus as shown in Figs 10, 12.

Females differ from males in the following: a) pronotum slightly wider but nearly as long as male (LP/WP 0.8-0.81); b) pronotum imperfectly subglobular and slightly sulcate, and c) elytra imperfectly subovate (LE/WE 1.14- 1.25), longer and wider (WE/WP 1.59–1.6, LE/LP 2.45–2.5) than in male, widest before middle; d) ventrite 1 flattened or slightly convex on disc. Metasternum and Ventrite 1 flattish. Otherwise, female similar to the male.

#### Etymology.

The specific epithet is named after Reagan Joseph Villanueva (Davao, Philippines) for his great contribution in the advancement of insect studies in the Philippines, particularly the order Odonata. Invariable genitive.

#### Distribution.

**Figure 22.**
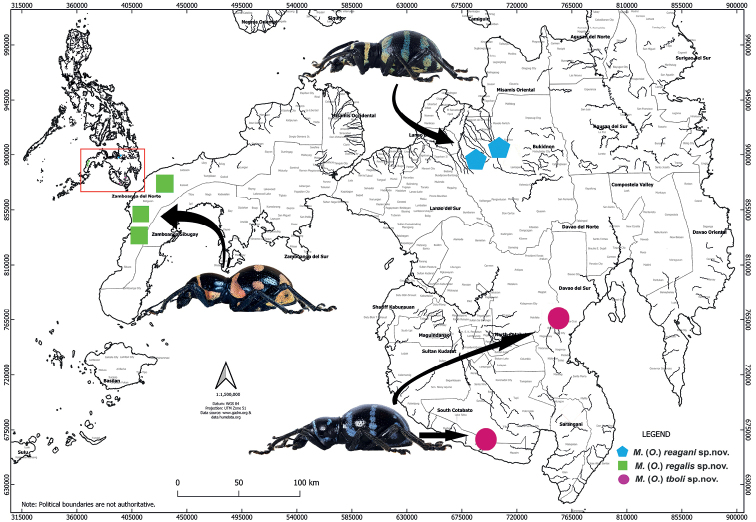
Distribution of Metapocyrtus (Orthocyrtus) spp. on Mindanao Island, Philippines.

Metapocyrtus (Orthocyrtus) reagani sp. nov. is known from a restricted area of Bukidnon Province (Fig. 22).

## Supplementary Material

XML Treatment for Metapocyrtus (Orthocyrtus) regalis

XML Treatment for Metapocyrtus (Orthocyrtus) tboli

XML Treatment for Metapocyrtus (Orthocyrtus) reagani
